# Efficacy and Safety of Tongning Gel for Knee Osteoarthritis: A Multicentre, Randomized, Double-Blinded, Parallel, Placebo-Controlled, Clinical Trial

**DOI:** 10.1155/2020/8707256

**Published:** 2020-06-11

**Authors:** Ye Zhao, Zhi Bi Shen, Ji Rong Ge, Wen Gang Liu, Jun Xing Yang, Cheng Jian He, Min Lu, Lin Shen, Hong Yin, Yong Qiang Chen, Zhi Bin Li, Qing Sun, Li Ming Xie, Wei An Yuan, Yu xin Zheng, Hong Sheng Zhan

**Affiliations:** ^1^Shuguang Hospital Affiliated to Shanghai University of Traditional Chinese Medicine, Shanghai 201203, China; ^2^Fujian Institute of Traditional Chinese Medicines, Fuzhou 350003, China; ^3^Guangdong No. 2 Hospital of Traditional Chinese Medicine, Guangzhou 510095, China; ^4^No. 1 Hospital Affiliated to Guangzhou University of Traditional Chinese Medicine, Guangzhou 510405, China; ^5^Hubei Hospital of Traditional Chinese Medicine, Wuhan 430061, China; ^6^No. 1 Hospital Affiliated to Hunan University of Traditional Chinese Medicine, Changsha 410021, China; ^7^Union Hospital Affiliated to Tongji Medical College, Huazhong University of Science and Technology, Wuhan 430022, China; ^8^Nanjing Hospital of Traditional Chinese Medicine, Nanjing 210022, China; ^9^Shanghai Hospital of Traditional Chinese Medicine, Shanghai 200071, China; ^10^Hospital Affiliated to Shanxi University of Traditional Chinese Medicine, Xianyang 712000, China; ^11^No. 1 Hospital Affiliated to Tianjin University of Traditional Chinese Medicine, Tianjin 300193, China; ^12^Guang'anmen Hospital Affiliated to the China Academy of Traditional Chinese Medical Sciences, Beijing 100053, China

## Abstract

**Objective:**

To evaluate the efficacy and safety of Tongning Gel (TNG) compared to placebo-controlled (PC) for knee osteoarthritis (KOA).

**Methods:**

A multicentre, randomized, double-blinded, parallel, placebo-controlled, clinical trial was performed in 576 patients (432 patients in the TNG group, 144 patients in the PC group), and 1 in the experimental group withdrew due to nonuse of drug. Patients were randomized to receive TNG or PC applied to knee skin at 3g per time, 2 times per day, which lasted for 3 weeks. The Western Ontario and McMaster Universities Arthritis Index (WOMAC) pain score was used to evaluate the primary efficacy of TNG and WOMAC stiffness and physical function and total scores were used to evaluate the secondary efficacy of TNG. All participants who received at least one dose of study drug were included in the safety analysis. This trial has been registered in Chinese Clinical Trial Registry (no. CTR20131276).

**Results:**

Primary efficiency outcome: there were significant differences in the decreased value of WOMAC pain score between two groups (*P* < 0.05), and the decreased value of WOMAC pain score in the TNG group were better than those in the PC group (*P* < 0.05). Secondary efficiency outcome: the WOMAC total score, WOMAC stiffness score, WOMAC physical function score, and the decrease of the above indexes of the two groups of patients after treatment were statistically significant (*P* < 0.05), and the improvement of the above indexes in the TNG group was better than that of the PC group (*P* < 0.05). *Safety Evaluation*. A total of 42 adverse events were reported by 29 patients: 25 adverse events reported by 16 patients (3.71%) in the experimental group and 17 adverse events were reported by 13 patients (9.03%) in the control group. And 8 adverse reactions were reported by 6 patients including 2 adverse reactions by 2 patients (0.46%) in the experimental group and 6 adverse reactions by 4 patients (2.78%) in the control group. Two cases of significant adverse events occurred in the experimental group. Both groups had one serious adverse event, respectively, which were not relevant to the intervention.

**Conclusion:**

These results of the trial demonstrate that TNG is superior to placebo in the treatment of patients with KOA, and TNG can improve other symptoms of KOA, such as stiffness and physical function. TNG is safe for the treatment of knee osteoarthritis as a whole.

## 1. Introduction

Knee osteoarthritis (KOA) is a highly prevalent joint disease and a leading cause of pain, impaired function, and disability which brings pressure and economic burden to society and family [1, 2]. In China, 3% of Chinese have osteoarthritis, mostly Knee OA. About 60% of people over the age of 55 have radiographic evidence of KOA and the incidence rate of the aged over 65 is 85% [3, 4]. With the advent of an aging society, the incidence of KOA has been increasing year by year, seriously affecting the quality of life and health of elderly patients. To date, the exact cause of KOA is not completely clear, and the risk factors for its pathogenesis include heredity, a history of previous injury, being women, obesity, and age [5]. There is no specific efficiency treatment for KOA, and the main objectives in the management of KOA are only to relieve pain or swelling, improve mobility of the joint, and minimize disability. Recently, with further research and clinical studies, although treatments for KOA including physical therapy, nonsteroidal anti-inflammatory drugs (NSAIDs), knee braces, opioids, intra-articular corticosteroid injections, and surgical treatments are commonly used to relieve symptoms, most treatments have been questioned in terms of the safety and efficiency for KOA [6, 7]. Thus, to find a safe, convenient, and effective treatment for KOA is urgent and significant. In China, Traditional Chinese Medicine (TCM) has received more and more attention in the treatment of KOA [8]. The distinctive TCM external therapy has remarkable curative effect and at the same time can reduce the gastrointestinal side effects of NSAIDs, which are the hotspots of current focus in the field. Modern research believes that TCM external therapy can improve the blood circulation of the knee joint, promote the metabolism of inflammatory mediators, relieve local muscle spasm, and improve pain [9, 10]. Based on the theory of TCM, Tongning Gel (TNG) originates from imperial physicians' formula “Gu Shang Teng Yao” (Remedies for Traumatological Conditions) in the Qing Dynasty and it is modified by Shu Chun Sun, chief physician and chief researcher in China Academy of Chinese Medical Science. In addition, the previous results of animal experiments and clinical trial have shown that the treatment of TNG for KOA is effective and safe. But with the lack of a large sample multicentre clinical trial, further clinical evidence on TNG in the treatment of KOA is needed. This clinical trial was designed to evaluate the efficacy and safety of TNG in patients with KOA further in a multicentre, randomized, double-blinded, parallel placebo-controlled, clinical trial.

## 2. Materials and Methods

### 2.1. Design and Randomization

To study the efficiency and safety of TNG in patients for KOA, we conducted a multicentre, randomized, double-blind, placebo-controlled clinical trial. The TNG group and the PC group in the clinical trial were allocated randomly in a ratio of 3 : 1 by using computer generated numbers. The randomized treatment assignments were sealed in opaque envelopes and opened individually for each patient who agreed to be in the study. The nurse, who had no role in the design and conduct of the study, prepared the envelopes. The researchers were out of touch with drugs, and the research pharmacy gave participants the same vials with TNG and PC directly, whose appearance and smell were identical. Patients in TNG group and PC group were both treated with TNG and PC, respectively, twice a day. At baseline, immediately after intervention, 1 week, 2 weeks, and 3 weeks, the Western Ontario and McMaster Universities Arthritis Index (WOMAC, range from 0 to 2400) was used as outcomes to evaluate. This trial has been registered in Chinese Clinical Trial Registry (No. CTR20131276).

### 2.2. Participants

Patients were recruited from Shuguang Hospital Affiliated to Shanghai University of Traditional Chinese Medicine, Fujian Institute of Traditional Chinese Medicine, Guangdong No. 2 Hospital of Traditional Chinese Medicine, No. 1 Hospital Affiliated to Guangzhou University of Traditional Chinese Medicine, Hubei Hospital of Traditional Chinese Medicine, No. 1 Hospital Affiliated to Hunan University of Traditional Chinese Medicine, Union Hospital Affiliated to Tongji Medical College, Huazhong University of Science and Technology, Nanjing Hospital of Traditional Chinese Medicine, Shanghai Hospital of Traditional Chinese Medicine, Hospital Affiliated to Shanxi University of Traditional Chinese Medicine, No. 1 Hospital Affiliated to Tianjin University of Traditional Chinese Medicine, and Guang'anmen hospital affiliated to the China Academy of Traditional Chinese Medical Sciences, Chinese Academy of Traditional Chinese Medicine between July 2012 and August 2013.

Inclusion criteria were as follows: (1) patients with unilateral KOA who meet the western diagnostic criteria and the standard of TCM syndrome differentiation; (2) age range from 40 to 70 years, including 40 and 70 years, male or female; (3) KL classification ≤ grade 3; (4) WOMAC pain score ≤ 350; (5) willing to participate in this study and signing the informed consent.

Exclusion criteria were as follows: (1) women who are breastfeeding, pregnant, or planning to become pregnant; (2) patients with allergic constitution or allergy to TNG; (3) patients with transient synovitis, slipped femoral capital epiphysis, bone tuberculosis, bone tumor, and pigmented villonodular synovitis; (4) patients with severe primary diseases such as cardiovascular, cerebrovascular, and hematopoietic systems, mental diseases, and abnormal liver and kidney functions; (5) patients who received intra-articular injection within 6 months prior to the study; (6) patients used disease-improving drugs and cartilage protectors within 6 months prior to the study; (7) patients were treated with corticosteroids, acupuncture, or physical therapy 1 week before treatment; (8) the skin of patients on the medication sites being damaged; (9) patients with long-term use of other drugs that affect the efficacy and safety judgment and have comprehensive treatment; (10) patients who are difficult to be evaluated in terms of the efficacy and safety of TNG for their critical conditions; (11) patients who participated in other study within 3 months before this study; (12) researchers considered patients inappropriate to be included.

### 2.3. Interventions

Drug used in the TNG group were as follows: Tongning Gel, main ingredients are Cu Yan Hu Suo (Vinegar Rhizoma Corydalis), Chuan Xiong (Rhizome Chuanxiong), Wei Ling Xian (Radix Clematidis), Shen Jin Cao (*Lycopodium clavatum*), Dong Bei Tou Gu Cao (Garden Balsam Stem in northeast China), Lu Lu Tong (Fructus Liquidambaris), Hai Tong Pi (*Erythrina indica* Lam), Fang Feng (Radix Sileris), Hua Jiao (Si Chuan Pepper), and Niu Xi (Radix *Achyranthis bidentatae*). The specification of TNG is 15 g per pieces, stored in the dry, dark, and room temperature condition. Jiangsu Kangyuan Pharmaceutical Co. Ltd. provided TNG (Batch NO. 20120301, valid for 15 months) for this study.

Drug used in the PC group were as follows: main ingredients are placebo starch materials to simulate TNG. The specification of placebo is 15 g per pieces, stored in the dry, dark, and room temperature condition. Jiangsu Kangyuan Pharmaceutical Co. Ltd. provided TNG (Batch NO. 20120301, valid for 15 months) for this study.

Patients in the TNG group and the PC group were given the TNG and PC, respectively, and both applied to knee skin at 3 g per time, 2 times per day, which lasted for 21 days. And patients were asked to use their medication at least eight hours apart during the day.

### 2.4. Outcomes

#### 2.4.1. Primary and Secondary Efficacy

WOMAC pain score was used to evaluate the primary efficacy of TNG and WOMAC stiffness, and physical function and total score were used to evaluate the secondary efficacy of TNG. These outcomes were evaluated at 1 week, 2 weeks, and 3 weeks, respectively. Comparing the decreased values from baseline between group, it is evaluated validly that the TNG group is better than the PC group at the endpoint of treatment (3 weeks).

#### 2.4.2. Safety Evaluation

TNG was evaluated the safety by the following criteria: (1) blood routine (red blood cell, neutral blood cell, lymphocyte, haemoglobin, platelet, urine routine, and stool routine); (2) liver function (ALT, AST, ALP, r-GT, and TBIL), and renal function (BUN, Cr); (3) electrocardiogram (ECG); (4) X-ray examination (before treatment); (5) adverse events and adverse reactions.

#### 2.4.3. Statistical Analysis

All statistical analyses were programmed using SAS 9.2 version statistical analysis software. All statistical tests used a two-sided test, and a *P* value of less than or equal to 0.05 is considered statistically significant (except for special instructions). The confidence interval uses 95% confidence. Baseline data were analyzed by full analysis set. All efficacy indicators were analyzed according to the full analysis set (FAS). The safety analysis was performed using the safety analysis set (SS). The description of the quantitative indicator will be expressed by the mean and standard deviation (mean ± SD). The description of the classification indicators is based on the number of cases and percentages. The enrolment analysis of each centre and the analysis of dropout rate are mainly based on descriptive analysis. If necessary, the total dropout rate of the two groups and the dropout due to adverse events will be compared by *χ*^2^ test or Fisher exact probability. Use *t*-test or *χ*^2^ test to compare demographic data with other baseline values to measure the balance between the two groups. The variance analysis method was used to evaluate the effectiveness index. Since this study is a multicentre clinical trial, the central effect on the efficacy index will be considered in this analysis. Comparisons of posttreatment declines between the primary efficacy measures were treated with a random effects model with adjustments for centre, gender, age, and baseline. Descriptive statistical analysis is the main cause of security analysis. If necessary, Fisher's exact probability method is used to compare the incidence of adverse events.

#### 2.4.4. Equality Control

In order to ensure the baseline balance between the two groups of patients, the patients were prevented from exercising knee joints during the whole study period, such as climbing, running, and climbing stairs. A supervisor was sent during the study to monitor the quality of research in all centres during the study. Each research centre is staffed with a full-time Clinical Research Coordinator (CRC). At the same time, in the implementation of the trial, two independent third-party clinical research quality audits, the audit results have shown that the entire clinical study met the requirements of the Good Clinical Practice (GCP).

## 3. Results

### 3.1. Patients

**Figure 1:**
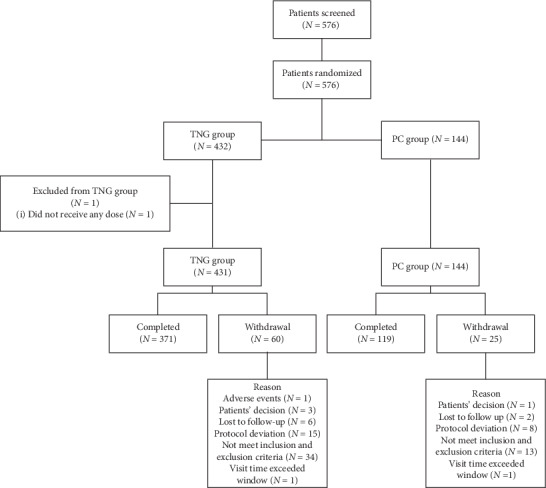
Screening, randomization, and completion evaluations from the baseline to 3-week follow-up.

Between July 2012 and November 2012, a total of 576 patients who passed the screening test were enrolled and randomly assigned with the ratio of 3 : 1 to each group (432 patients in the TNG group, 144 patients in the PC group), and 1 in the experimental group withdrew due to nonuse of drug. Both groups reported outcomes at 1 week, 2 weeks, and 3 weeks after intervention. In the safety analysis set (SS), 575 patients including 431 patients in the TNG group and 144 patients in the PC group who used the assigned TNG at least once were asked if they had experienced any adverse effects. In the full analysis set (FAS), 575 patients who used at least one dose of the received TNG and provided data for evaluating the primary efficacy endpoint included 431 patients in the TNG group and 144 patients in the PC group. In the per protocol set (PPS), 491 patients who completed the treatment included 372 patients in the TNG group and 119 patients in the PC group. This study used intention-to-treatment analysis (Figure 1).

### 3.2. Baseline

As shown in Table 1, there was no statistically significant difference between groups with respect to demographic data (gender, age, height, and weight) and vital signs (heart rate, respiratory rate, and blood pressure) (*P* > 0.05). In terms of duration of Knee OA, location of the disease KL classification, history of treatment and drug allergy, and other diseases, no significant difference was observed between two groups (*P* > 0.05). And the baseline WOMAC score including pain, stiffness, and function and total score were also reasonably comparable between TNG group and PC group (*P* > 0.05).

### 3.3. Efficacy Results

#### 3.3.1. Primary Efficacy Outcomes

**Figure 2:**
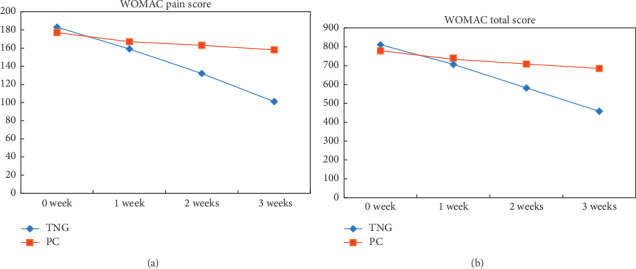
Mean change of the WOMAC pain score, (a) and WOMAC total score. (b) The means of outcomes are shown for the TNG group (diamond) and the PC group (square). Measurements were observed at baseline, 1 week, 2 weeks, and 3 weeks after intervention. WOMAC pain score ranges from 0 to 500, and WOMAC total score ranges from 0 to 2400.

As shown in Tables 2 and 3 and Figure 2, the mean WOMAC pain score declined gradually over time in both groups. In 1 week, no statistically significant difference was observed between groups (*P*=0.2074). And statistically significant differences were observed in 2 weeks (*P* < 0.001) and 3 weeks (*P* < 0.001) between groups. The decreased WOMAC pain value improved gradually over time in both groups. Compared to the control group, the experimental group showed more decline from baseline, and between-group difference of decreased pain value was statistically significant at each time point (*P* < 0.001).

#### 3.3.2. Secondary Efficacy Outcomes

As shown in Tables 2 and 3 and Figure 2, the mean WOMAC stiffness, physical function, and total score also declined gradually over time in both groups. There was no statistically significant difference found between groups (*P* > 0.05) after 1-week intervention. However, in 2 weeks and 3 weeks, statistically significant differences were observed (*P* < 0.05). The decreased WOMAC stiffness, physical function, and total value improved gradually over time in both groups. The experimental group values decreased more than the control group values from baseline, and no significant difference between groups were observed at each time point (*P* < 0.001).

#### 3.3.3. Adverse Events

As shown in Table 4, a total of 42 adverse events were reported by 29 patients: 25 adverse events reported by 16 patients (3.71%) in the experimental group and 17 adverse events by 13 patients (9.03%) in the control group. And 8 adverse reactions were reported by 6 patients including 2 adverse reactions by 2 patients (0.46%) in the experimental group and 6 adverse reactions by 4 patients (2.78%) in the control group. Two cases of significant adverse events occurred in the experimental group. Both groups had one serious adverse event respectively which was not relevant to the intervention.

#### 3.3.4. Drug-Use Combination

A total of 96 patients who had drug-use combination were reported in 575 patients: 73 patients (16.94%) in the experimental group and 23 patients (15.97%) in the control group. Between groups, no statistically significant difference was found (*P* > 0.05).

## 4. Discussion

This is a multicentre, random, double-blind, parallel, placebo-controlled clinical trial to provide the evidence that TNG has efficiency and safety for the treatment of patients with KOA.

Modern medical research on KOA has not clearly clarified its pathogenic mechanism [11]. The majority consider that degenerative changes and destruction of articular cartilage are the basic pathological changes, while bone hyperplasia and subchondral bone sclerosis are secondary pathologic changes which can involve the synovial membrane of the joint and induce synovitis and thus acute inflammatory reaction [12]. The theory of TCM categorizes KOA into impediment-related conditions. It is believed that contributing factors of this condition include deficiency of qi and blood, deficiency of liver and kidney, and wind, cold, and dampness affecting the bone. These factors may cause qi and blood to stagnate within meridians, and thus resulting in pain [13].

TNG originates from imperial physicians' formula “Gu Shang Teng Yao” (Remedies for Traumatological Conditions) in the Qing Dynasty. In Chinese medical theory, this formula acts to move blood, regulate qi, alleviate pain, remove wind, unblock meridians, and resolve dampness. Some studies have concluded that taking Traditional Chinese Medicine reinforces kidney, activates blood, inhibits the formation of inflammatory factors, degrades cartilage matrix and the apoptosis of chondrocytes, and promotes the proliferation of chondrocytes [14]. The previous preclinical animal experiments of TNG and the outcomes confirmed that TNG can improve the blood supply to the bone and accumulate dead bone resorption as well as new bone formation in the rabbit KOA model. TNG also increases the models' cartilage moisture content and cartilage glucuronic acid, promotes the synthesis of chondrocyte matrix and repair of articular cartilage, eliminates synovial congestion and swelling, and promotes the absorption of joint inflammation. In terms of relieving pain, TNG increased the pain threshold of mice. Meanwhile, no obvious toxic and side effects, skin irritation, and skin allergy were observed in the acute and long-term toxicity test by skin application. Furthermore, the previous Phase II clinical trial has shown that the TNG group are better than the PC group both in the change of WOMAC pain score and up-and-down stairs pain score. Only 1 adverse reaction which showed pruritus might be relevant to TNG, and the safety of the two groups was good and no serious adverse events occurred.

The findings of this trial have shown that TNG does have good efficacy and safety in patients with KOA. Changes in the main efficacy index WOMAC pain score show pain relief in both groups after treatment, and the decreased WOMAC pain score index in the TNG group is better than the PC group which showed that TNG can significantly improve the knee pain in patients. Similarly, we found that the secondary efficacy index including WOMAC stiffness, function, and total score improved in both groups after treatment, and the decreased index in the TNG group is better compared with PC group, which showed that TNG could improve other symptoms of knee osteoarthritis as a whole in addition to being effective in knee pain.

With regard to the safety compared with the PC group, TNG for patients with KOA is safer. The incidence of adverse events and adverse reactions in the TNG group was lower than that in the PC group, and one serious adverse event occurred in each group, which was judged to be unrelated to the drug. There were 2 adverse events in the TNG group: “dermatitis” and drug relationship were judged as “NK,” and “skin allergy” and drug relationship were judged as “suspicious,” considering that TNG is a topical preparation, so it is necessary to pay attention to the skin irritation of TNG.

Our study has several strengths and limitations to consider. Given the notion that the safety and efficiency of lifestyle interventions have been advocated in the patients with KOA due to low risk, the use of NSAIDS should be minimized on account of its adverse gastrointestinal reactions [15]. And the major strength of this trial is that TNG as a Traditional Chinese Medicine Gel has advantage of high absorption speed, high bioavailability, good biocompatibility, uniform texture, and being easy to spread and clean [16, 17]. The main limitation of this trial is that the chemical composition of TNG is unknown.

## 5. Conclusions

The results of this clinical trial demonstrate that TNG over placebo in terms of reducing joint pain, stiffness, and physical function in patients with KOA through 3 weeks of treatment is better, and TNG is safe for the treatment of Knee Osteoarthritis as a whole.

## Figures and Tables

**Table 1:** Baseline characteristics between experimental group and control group.

Variable	TNG (*n* = 431)	PC (*n* = 144)	*P* value
Gender, *n* (%)			1.0000
Male (%)	94 (21.81%)	31 (21.53%)	
Female (%)	337 (78.19%)	113 (78.47%)	
Age (years)	55.44 ± 8.16	54.44 ± 8.20	0.2035
Height (cm)	162.83 ± 6.56	162.43 ± 7.06	0.5323
Weight (kg)	61.73 ± 8.13	60.89 ± 8.77	0.2906
Heart rate (beats/min)	71.69 ± 7.99	71.26 ± 8.52	0.5787
Respiratory rate (beats/min)	18.52 ± 1.55	18.68 ± 1.87	0.3677
Systolic BP (mmHg)	123.23 ± 9.29	122.58 ± 10.23	0.4785
Diastolic BP (mmHg)	77.63 ± 6.70	76.95 ± 7.47	0.3370
Duration of KOA (months)	8.00 ± 21.00	6.00 ± 14.00	0.530
Location of the disease, *n* (%)			0.0129
Left knee	207 (48.03%)	55 (38.19%)	
Right knee	223 (51.74%)	86 (59.72%)	
Both knees	1 (0.23%)	3 (2.08%)	
Baseline WOMAC, mean ± SD			
Pain score	183.61 ± 64.86	177.36 ± 66.92	0.3215
Stiffness score	47.87 ± 31.53	46.76 ± 32.48	0.7165
Function score	579.88 ± 195.62	555.01 ± 202.97	0.1913
Total score	811.35 ± 272.16	779.13 ± 281.19	0.2230
KL classification, *n* (%)			0.738
Grade 0	18 (4.18%)	13 (9.03%)	
Grade 1	116 (26.91%)	32 (22.22%)	
Grade 2	246 (57.08%)	82 (56.94%)	
Grade 3	51 (11.83%)	17 (11.81%)	
History of treatment, *n* (%)			1.0000
No	430 (99.77%)	144 (100.00%)	
Yes	1 (0.23%)	0 (0.00%)	
Other disease: no. of patients (%)			0.7018
No	403 (93.50%)	133 (92.36%)	
Yes	28 (6.50%)	11 (7.64%)	
History of drug allergy: no. of patients (%)			0.6977
No	425 (98.61%)	141 (97.92%)	
Yes	6 (1.39%)	3 (2.08%)	

Plus-minus values are means ± SD unless otherwise noted. TNG = Tongning Gel; PC = placebo-controlled. WOMAC = the Western Ontario and McMaster Universities Arthritis Index. KL classification = Kellgren–Lawrence classification.

**Table 2:** Comparison of WOMAC scores between two groups before and after intervention.

	0 weeks			1 week			2 weeks			3 weeks		
	TNG (*n* = 431)	PC (*n* = 144)	*P* value	TNG (*n* = 431)	PC (*n* = 144)	*P* value	TNG (*n* = 431)	PC (*n* = 144)	*P* value	TNG (*n* = 431)	PC (*n* = 144)	*P* value
Pain	183.61 ± 64.86	177.36 ± 66.92	0.3215	159.63 ± 61.09	167.19 ± 65.53	0.2074	132.44 ± 57.51	163.13 ± 68.96	<0.0001	101.45 ± 58.94	158.03 ± 75.01	<0.0001
Stiffness	47.87 ± 31.53	46.76 ± 32.48	0.7165	42.45 ± 29.47	43.99 ± 31.86	0.5939	35.00 ± 26.41	41.90 ± 31.49	0.0188	27.67 ± 23.71	40.42 ± 31.74	<0.0001
Function	579.88 ± 195.62	555.01 ± 202.97	0.1913	505.09 ± 187.64	523.15 ± 202.33	0.3275	415.13 ± 176.35	504.24 ± 211.74	<0.0001	329.11 ± 183.62	486.81 ± 227.44	<0.0001
Total	811.35 ± 272.16	779.13 ± 281.19	0.2230	707.17 ± 261.09	734.33 ± 279.83	0.2890	582.58 ± 246.31	709.26 ± 293.42	<0.0001	458.23 ± 254.54	685.26 ± 315.79	<0.0001

Plus-minus values are means ± SD unless otherwise noted. Comparison between TNG group and PC group by ANOVA.

**Table 3:** Comparison of decreased value from baseline between two groups.

	Mean ± SD (95% CI)		Between group difference, mean ± SD (95% CI)	*P* value
	TNG (*n* = 431)	PC (*n* = 144)		
Pain				
1 week	23.98 ± 26.24 (21.49, 26.46)	10.17 ± 23.42 (6.32, 14.03)	13.80 ± 2.46 (8.97, 18.64)	＜0.001
2 weeks	51.16 ± 40.27 (47.35, 54.97)	14.24 ± 32.57 (8.87, 19.60)	36.93 ± 3.70 (29.65, 44.20)	＜0.001
3 weeks	82.16 ± 54.24 (77.02, 87.29)	19.33 ± 47.85 (11.45, 27.22)	62.82 ± 5.07 (52.86, 72.79)	＜0.001

Stiffness				
1 week	5.42 ± 8.99 (4.57, 6.27)	2.76 ± 9.28 (1.24, 4.29)	2.66 ± 0.87 (0.94, 4.37)	0.0015
2 weeks	12.87 ± 15.46 (11.41, 14.33)	4.85 ± 11.63 (2.94, 6.77)	8.02 ± 1.41 (5.26, 10.78)	＜0.001
3 weeks	20.19 ± 18.76 (18.42, 21.97)	6.34 ± 16.09 (3.69, 8.99)	13.85 ± 1.75 (10.43, 17.28)	＜0.001

Physical function				
1 week	74.79 ± 71.12 (68.05, 81.52)	31.86 ± 58.40 (22.24, 41.48)	42.93 ± 6.56 (30.04, 55.81)	＜0.001
2 weeks	164.74 ± 121.07 (153.28, 176.20)	50.77 ± 91.85 (35.64, 65.90)	113.97 ± 11.02 (92.33, 135.61)	＜0.001
3 weeks	250.77 ± 162.20 (235.41, 266.13)	68.19 ± 137.81 (45.49, 90.89)	182.58 ± 15.06 (153.00, 212.16)	＜0.001

Total				
1 week	104.18 ± 97.59 (94.94, 113.42)	44.80 ± 84.95 (30.81, 58.79)	59.38 ± 9.10 (41.50, 77.27)	＜0.001
2 weeks	228.77 ± 166.06 (213.05, 244.50)	69.86 ± 129.27 (48.57, 91.16)	158.91 ± 15.18 (129.10, 188.72)	＜0.001
3 weeks	353.12 ± 224.1 7 (331.90, 374.35)	93.87 ± 196.14 (61.56, 126.18)	259.25 ± 20.94 (218.13, 300.38)	＜0.001

Comparison between TNG group and PC group by ANOVA.

**Table 4:** Comparison of adverse events and incidence of adverse reactions between two groups.

	TNG (*n* = 431)		PC (*n* = 144)		*P* value
	Frequency	Case (％)	Frequency	Case (％)	
Adverse event	25	16 (3.71)	17	13 (9.03)	*P*=0.0158
Adverse reaction	2	2 (0.46)	6	4 (2.78)	*P*=0.0370
Significant adverse event	2	2 (0.46)	0	0 (0.00)	*P*=1.0000
Serious adverse event	1	1 (0.23)	1	1 (0.69)	*P*=0.4385

Comparison between TNG group and PC group by Fisher's exact test.

## Data Availability

The data including subject information and the efficacy and safety data used to support the findings of this study have not been made available because in compliance with requirements and regulations for the registration of new drugs in China, all data have been submitted to the National Drug Review Center, for privacy protection, and business competition detailed data are generally not disclosed to the public, and unless major events occur after the drug is on the market, relevant data will be made public according to the law.
